# Improving the Thermal Stability of the Fine-Grained Structure in the Cu-15Ni-8Sn Alloy during Solution Treatment by the Additions of Si and Ti

**DOI:** 10.3390/ma16031252

**Published:** 2023-02-01

**Authors:** Chao Zhao, Daoxi Li, Xiaotao Liu, Minghan Sun, Zhi Wang, Zongqiang Luo, Weiwen Zhang

**Affiliations:** 1 School of Materials Science and Engineering, Huazhong University of Science and Technology, Wuhan 430074, China; 2 School of Mechanical and Automotive Engineering, South China University of Technology, Guangzhou 510640, China; 3 Department of Mechanical and Energy Engineering, Shaoyang University, Shaoyang 422004, China

**Keywords:** Cu-15Ni-8Sn alloys, grain size, solution treatment, microalloying, microstructural evolution

## Abstract

Grain refinement has been found to be an effective method for simultaneously enhancing strength and toughness. To avoid the sharp coarsening of grains in Cu-Ni-Sn alloys during solution treatment and thereby overcoming the tradeoff between strength and ductility, this work attempted to modify the composition and improve the thermal stability of the fine-grained structure in Cu-Ni-Sn alloys. The grain growth behavior during a solution treatment of the Cu-15Ni-8Sn alloys with/without Si and Ti additions was systematically investigated. The result reveals that compared to the grain size of 146 μm in the based alloy (without trace additions) after solution processing at 1073 K for 2 h, the fine-grained structure with a size below 20 μm is maintained owing to the benefit from Si and Ti addition. It was observed that the addition of Si and Ti offer the inhibition effect on the dissolution of the γ phase and Ni_16_Si_7_Ti_6_ particles after solution treatment. The grain boundary movement is severely hindered by these two aspects: the pinning effect from these particles, and the drag effect induced by additional solute atoms. Based on the analysis of grain growth kinetics, the activation energy of grain growth is increased from 156 kJ/mol to 353 kJ/mol with the addition of Si and Ti.

## 1. Introduction

Cu-15Ni-8Sn alloys possess excellent mechanical properties and excellent stress relaxation resistance, as well as excellent wear resistance and corrosion resistance, being considered as promising beryllium–copper substitute materials [1,2]. These high-performance copper alloys have great potential applications in the electronic and electrical industries, and simultaneously show wide prospects for major equipment involving aircraft landing gear, heavy loaded vehicles, and marine engineering equipment [3,4,5]. However, the trade-off between strength and ductility of the alloys is still the critical problem for meeting the increasing performance demands of key components in aerospace, mechanical systems, and the oil and gas industries. For example, after the commonly used pre-cooling deformation–aging treatment of the alloy, its strength can reach up to 1400 MPa, but its elongation rate is only ~1% [6]. One essential approach is to improve the plasticity of the alloy while maintaining its strength.

Grain refinement is commonly deemed an effective method to improve plasticity in polycrystalline metallic materials. Our previous research obtained the fine-grain structure in the Cu-15Ni-8Sn alloy via the hot extrusion process, effectively improving the elongation of the as-extruded alloy to more than 30% [7,8,9]. On the other hand, the Spinodal decomposition and ordering strengthening could not completely occur in the as-extruded alloy due to the absence of the subsequent solution and aging treatments, so it failed to reap the full performance advantages of the Cu-15Ni-8Sn alloy [10]. High temperature solution treatments are able to promote the formation of supersaturated solid solutions, providing a chemical driving force for Spinodal decomposition and ordering transformation [11,12], but they often lead to uncontrollable grain coarsening, encroaching on the superior ductility induced by the fine-grain structure via the hot extrusion process. A large number of studies have shown that the addition of trace elements can result in the development of the drag effect on solid solution atoms and the pinning effects on the insoluble second phase, which shows an important role of the suppression of grain boundary migration during solution treatment [13,14,15,16]. At present, a thorough investigation of the effect of trace elements on the grain growth kinetics and the thermal stability of grains is lacking and is critical to seek a way to realize the synergy of superior strength and high ductility of the Cu-15Ni-8Sn alloy.

In our previous research, the Cu-15Ni-8Sn-0.3Si-0.1Ti alloy was found to be of the optimal composition, as it showed a fine-grained structure even after solution treatment and thereby gained excellent comprehensive mechanical properties [7,8,17]. Accordingly, in the present work, a comprehensive comparison of the grain growth behavior between the Cu-15Ni-8Sn alloy with and without Si and Ti addition has been studied in order to uncover the effect of trace elements (for instance Si and Ti) in the thermal stability of the fine-grained structure during solution treatment. The microstructural features of the based and modified alloys during solution treatment, involving grain morphology and the size and distribution of secondary phase, were investigated in detail. The influences of trace additions on the thermal stability of grains were discussed from the perspective of the drag effect on solute atoms and the pinning effect on secondary particles. The information gained on grain growth behavior would help to maintain the grain refinement and further lay a foundation for the high ductility of the Cu-15Ni-8Sn alloy. 

## 2. Materials and Methods

The Cu-15Ni-8Sn-based alloy and Cu-15Ni-8Sn-0.3Si-0.1Ti modified alloy were prepared by an intermediate frequency induction furnace. The actual chemical compositions of the as-cast materials determined by optical emission spectroscopy (OES) are listed in Table 1. The cast ingots were homogenization-treated at 1113 K for 8 h and subsequently hot-extruded to a rod with a diameter of 12 mm at 1223 K. The extruded rods were cut and annealed at 1073 K, 1093 K, and 1113K for 0.5 to 2 h, followed by quenching in water. 

The microstructure of the annealed samples was characterized by optical microscopy (OM), scanning electronic microscopy (SEM) equipped with energy dispersive X-ray spectroscopy (EDS), and transmission electron microscopy (TEM). The mean grain size was obtained from at least 10 random fields of each specimen by the linear intercept method based on the observation with a LEICA/DMI 5000 M optical microscope. The specimens for OM and SEM observations were prepared by polishing and then etching in a solution of 5 g FeCl_3_ + 10 mL HCl + 100 mL H_2_O. The TEM observation was carried on a FEI TECNAI G2 S-TWIN F20 transmission electron microscope. TEM samples were prepared via the twin jet electro-polishing method in 30% nitric acid and 70% methanol held at 248 K. The hardness measurement was performed on a Vickers microhardness tester using a load of 100 gf and a dwelling time of 10 s. The average values of at least 5 measurements were used to establish the hardness data. The thermal stability of the fine-grained structure in the alloys was assessed through the above microstructural observations.

## 3. Results

**Figure 1:**
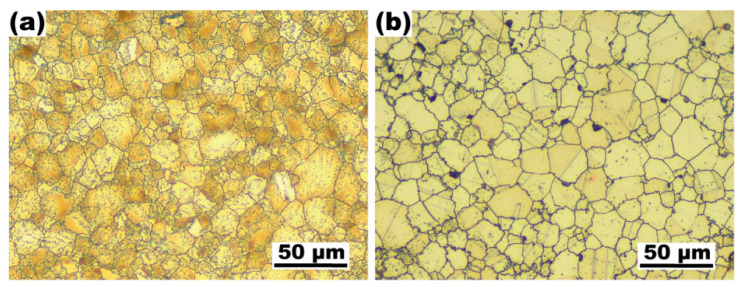
Representative OM images of as-extruded Cu-15Ni-8Sn-0.3Si-0.1Ti (**a**) and Cu-15Ni-8Sn (**b**) alloys.

Figure 1 shows the optical images of as-extruded Cu-15Ni-8Sn-0.3Si-0.1Ti and Cu-15Ni-8Sn alloys before the solution treatment. The average grain size of the alloy with additions of Si and Ti (12.4 μm) is slightly smaller than that of the based alloy (15.4 μm) owing to the formation of fine second phase particles [17].

**Figure 2:**
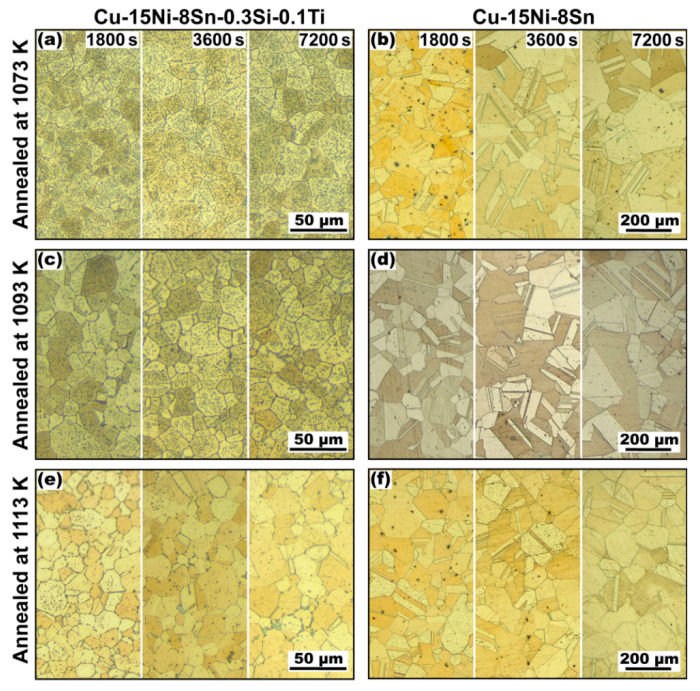
Representative optical images of the microstructure of the studied materials after annealing in different conditions: (**a**,**c**,**e**) Cu-15Ni-8Sn-0.3Si-0.1Ti; (**b**,**d**,**f**) Cu-15Ni-8Sn.

**Figure 3:**
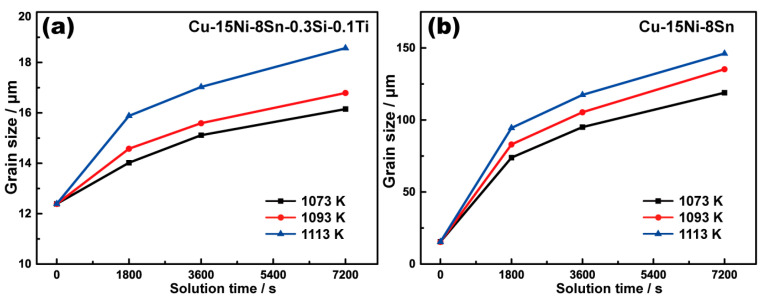
Average grain sizes of the Cu-15Ni-8Sn-0.3Si-0.1Ti (**a**) and Cu-15Ni-8Sn (**b**) alloys after different solution processes.

Figure 2 demonstrates a series of representative OM micrographs of Cu-15Ni-8Sn and Cu-15Ni-8Sn-0.3Si-0.1Ti alloys annealed at 1073 K to 1113 K for different time periods. The relevant average grain sizes are shown in Figure 3. It is evident that the grain size is increased with the increment increase in solution temperature and time, and a more rapid grain growth is induced at a higher temperature. Compared with the excessive grain growth in the Cu-15Ni-8Sn alloy, the velocity of grain growth in the modified alloy is much slower. Even after it was in the solution at 1113 K for 2 h, the increment in grain size in the modified alloy is only about 6 μm (from 12.4 μm to 18.6 μm), while it is measured as 130.7 μm in the based alloy (from 15.4 μm to 146.1 μm). 

It can be found from Figure 2a,c,e that the amount of second phase particles in the modified alloy is decreased with the prolongation of the solution time, and the dissolution rate of the second phase becomes faster with a higher solution temperature. After being in the solution at 1113 K for 2 h, only a few large-sized particles survive at the grain boundaries (Figure 2e). This positive relationship between the dissolution degree of the second phase particles and the grain growth rate in the modified alloy indicates that the existence of the second phase may be responsible for restraining the grain growth in the alloy.

For a quantitative comparison of the tendency of grain growth during annealing for 2 h, the grain growth factor (*G_f_*), defined as (*d_f_* − *d*_0_)/*d*_0_ [18] (where *d_f_* and *d*_0_ are the final and the initial grain sizes, respectively) is calculated and listed in Table 2. The value of *G_f_* of the based alloy is about 20 times bigger than that of the modified alloy, indicating a distinct suppression of the grain growth in the modified alloy due to the addition of Si and Ti.

**Figure 4:**
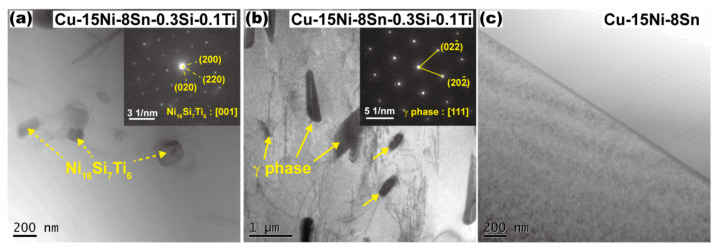
TEM images revealing the microstructures of Cu-15Ni-8Sn-0.3Si-0.1Ti (**a**) and Cu-15Ni-8Sn alloys (**b**,**c**) after annealing at 1093 K for 1 h. The inserts reveal the selected area diffraction patterns (SADP) of related particles.

In order to study the changes in the morphology and distribution of the second phase in the alloy with additions of Si and Ti under different solution conditions, further microstructural observation was conducted for the alloy in a 1093 K solution at different times. Figure 4 shows the microstructure of the based and modified alloys annealed at 1093 K for 1 h. According to the results of the selected area diffraction pattern (SADP), the precipitate phase is confirmed as a Ni_16_Si_7_Ti_6_ intermetallic compound with a size of ~200 nm [7] (Figure 4a). The rod-like γ phase with a DO3 structure was also confirmed by SADP analysis, which is consistent with J.C. Zhao’s results [11]. Meanwhile, no precipitates were observed in the based alloy after solution treatment, implying the improvement in thermal stability by the addition of Si and Ti.

**Figure 5:**
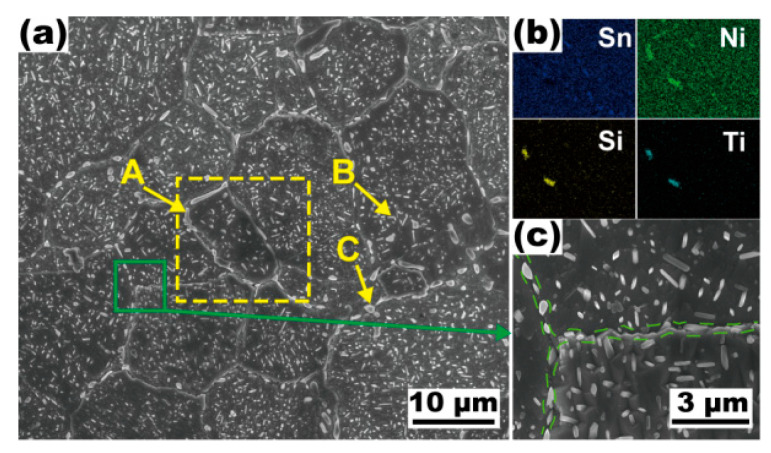
SEM micrographs of the Cu-15Ni-8Sn-0.3Si-0.1Ti alloy after annealing at 1093 K for 5 min. (**a**) Grain morphology in the alloy; (**b**) the EDS-mapping result corresponding to the yellow rectangle in (**a**); (**c**) enlarged view of the green domain in (**a**), small needle-like particles distributed along the grain boundaries are marked by the green dotted line.

Figure 5 shows the SEM images and energy dispersive surface scanning results of the 0.3Si-0.1Ti alloy after solution processing at 1093 K for 5 min. There are a large number of second phases formed with the grains and at grain boundaries. According to the results of the EDS analysis shown in Table 3, these particles are defined as Ni_16_Si_7_Ti_6_ and γ phases. Figure 5b shows the rod-like Ni_16_Si_7_Ti_6_ particles (~2.8 μm) distributed at the grain boundaries. It can be seen from the high magnification SEM image (Figure 5c) that small needle-like particles are distributed along the grain boundaries (as marked by the green dotted line), whose average major axis, minor axis, and aspect ratio are about 617 nm, 225 nm, and 2.8, respectively.

**Figure 6:**
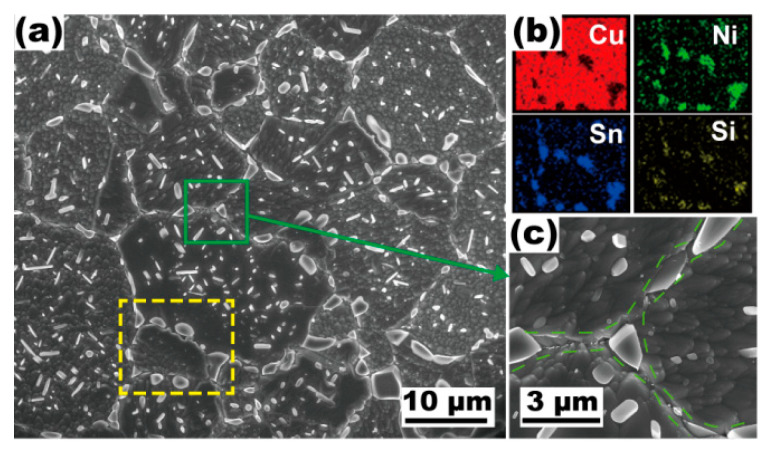
SEM micrographs of the Cu-15Ni-8Sn-0.3Si-0.1Ti alloy after annealing at 1093 K for 1 h. (**a**) Grain morphology in the alloy; (**b**) the EDS-mapping result corresponding to the yellow rectangle in (**a**); (**c**) higher magnification image of the green domain in (**a**), intergranular micron particles are highlighted by the green dotted line.

Figure 6 indicates the microstructural features of the modified alloy after solution processing at 1093 K for 1 h. A large amount of the Ni_16_Si_7_Ti_6_ and γ phases can still be observed in the alloy. Figure 6b shows micron particles are distributed around the grains, which implies the inhibiting effect of grain growth induced by these insoluble particles. The amount of fine needle-like second phase which survived after solution processing for 5 min is significantly reduced, while there are still densely distributed micron particles with an average size of 1.2 μm (Figure 6c).

**Figure 7:**
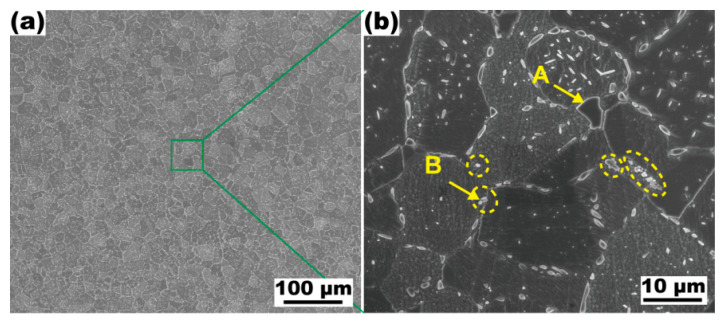
SEM micrographs of the Cu-15Ni-8Sn-0.3Si-0.1Ti alloy after annealing at 1093 K for 2 h. Intergranular micron particles are highlighted by the yellow dotted line.

Figure 7 shows the SEM image of the modified alloy after solution processing at 1093 K for 2 h. The fine-grained structure is still retained in the alloy despite solid solution processing for 2 h (Figure 7a). The amount of intragranular second phase is markedly reduced, while relatively more particles are distributed at grain boundaries (Figure 7b). According to the results of the EDS analysis shown in Table 4, these intergranular particles are of the Ni_16_Si_7_Ti_6_ and γ phases, indicating that these second phases have good thermal stability, and show indissolubility in the solution process at 1093 K. 

According to the TTT diagram summarized by J.C. Zhao and M.R. Notis [11], the Cu-15Ni-8Sn alloy was located in the so-called α single phase area with a temperature above 1073 K. As a result, the γ phase would be dissolved completely in this temperature window, which is consistent with the microstructure of the based alloy after solution processing at 1093 K for 1 h (Figure 4a). On the contrary, the existence of the γ phase in the modified alloy after solution indicates that the γ phase shows a higher thermal stability due to the addition of Si and Ti.

## 4. Discussion

### 4.1. Kinetics of Grain Growth of the Alloys with and without Additions

**Figure 8:**
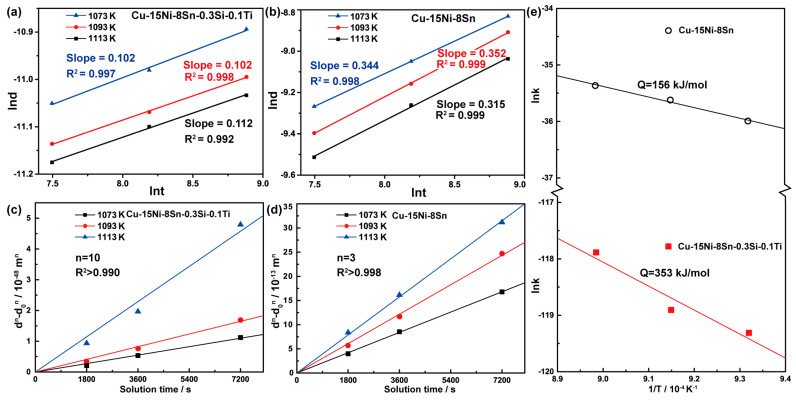
The grain growth kinetics analysis of the studied alloys during annealing treatment. (**a**,**b**) Linear fitted plots of *lnd*-*lnt*; (**c**,**d**) curve fit plots of Equation (1) used to estimate grain growth exponents; (**e**) grain growth exponents as a function of the reciprocal of absolute temperature used to calculate the grain growth activation energy.

The kinetics of grain growth can be deduced by analyzing the grain size as a function of time, which is depicted as the well-known parabolic kinetic model [19]:

(1) $$d^{n}-d_{0}^{n}=kt$$

where *d* is the instantaneous grain size, *d*_0_ is the initial grain size that can be considered as the grain size of as-extruded alloys, *t* is the solution holding time, *n* is a grain growth exponent, and *k* is a kinetic constant, which depends primarily on the temperature and grain boundary energy. As shown in Figure 8a,b, the value 1/*n* can be calculated as the slope of linear fitting plots of *lnd-lnt*. The results of the based and modified alloys are 10 and 3, respectively. The relationship between the grain size and solution time can be obtained by introducing the *n* value into Equation (1), which is shown in Figure 8c,d. The constant *k* has an Arrhenius form as [12,13]:

(2) $$k=k_{0}\exp(-Q/RT)$$

in which *k*_0_ is the pre-exponential constant and *Q* is the grain growth activation energy. *R* is the gas constant and *T* is the absolute temperature. The *k* values of these two alloys at different solution temperatures can be obtained from Figure 8c,d. By plotting semi-logarithmic plots of ln(*k*) versus 1/*T*, as demonstrated in Figure 8e, the value of the grain growth activation energy (*Q*) in the based and modified alloys can be measured from the slope of the plotted curves, which is 156 kJ/mol and 353 kJ/mol, respectively. More than twice the activation energy is required in the modified alloy to trigger grain growth, indicating that the higher thermal stability of the fine-grained structure is induced by the addition of Si and Ti.

Burke and Turnbull found that if the driving force of grain growth in pure metals under ideal conditions is mainly from the grain boundary curvature, the grain growth exponent (*n*) in Equation (1) is equal to 2 [20]. Fan and Chen found that when the grain growth is determined by the long-range atomic diffusion in the two-phase solid solution, the grain growth exponent (*n*) in Equation (1) is equal to 3 [21]. In the study of multi-component alloys, such as magnesium-based alloys, nickel-based alloys, iron-based alloys, and high-entropy alloys, the *n* value is approximately 3 [12,13], which is consistent with the *n* value of Cu-15Ni-8Sn. However, the higher value of the grain growth exponent (*n*) indicates that the grain growth dynamics in the alloy are also affected by other factors, which are often related to the solid solution atoms, the second phase, texture, etc. In this study, the role of trace elements in the modified alloy in increasing the value of *n* mainly involves the drag effect of solid solution atoms and the pinning effects of the second phase.

### 4.2. Effects of the Additions of Trace Elements on the Grain Growth

In order to explain the effects of the addition of Si and Ti, the grain boundary migration rate (*V_gb_*) was introduced which is related to the diffusion ability of the solute and the interaction between the grain boundary and solute atoms. It is assumed that the interaction energy between the grain boundary and one solute atom is *E*(*x*), and the diffusion coefficient of the solute atom perpendicular to the grain boundary is *D*(*x*). Both *E*(*x*) and *D*(*x*) are the functions of the distance *x* from the center of the grain boundary. When *V_gb_* is low, the atomic drag (α) can be expressed as [12]:

(3) $$\alpha =4N_{v}kT\int \frac{\sinh^{2}\left(E(x)/2kT\right)}{D(x)}dx$$

where *N_v_* is the number of solute atoms per unit volume, *k* is the Boltzmann constant, and *T* is the absolute temperature. According to Equation (3), the solute drag effect (*α*) shows an inversely proportional relationship to the diffusivity of solute atoms, as α∝1/D(x). Thus, *V_gb_* can be expressed as:

(4) $$V_{gb}=\frac{P}{\lambda +\alpha C_{0}}$$

where *P* is the driving force of grain growth, *C*_0_ is the concentration of solute in the alloy, and *λ* is a constant. Generally, *C*_0_ is considered as a constant in the same alloy. As a result, *V_gb_* is inversely proportional to *α*, equivalently there is a proportional relationship between *V_gb_* and *D*(*x*), indicating that the lower the diffusion ability of the solute atoms is, the stronger the drag effect is. The diffusion coefficients of each solute atom in pure copper are used for analysis (as shown in Table 5) [22]. Compared with the diffusion ability of Sn, the diffusion coefficients of Si and Ti in pure copper are much lower, enhancing the drag effect of solute atoms on grain boundaries. Based on microstructural observation, the concentration of Si (0.23 at%) dissolved in the modified alloy is about 3 times higher than that of Ti (0.06 at%), indicating that the addition of Si should play a dominant role in the drag effect.

The pinning effect of the second phase on grain boundary migration depends on the radius (*r*), shape, spacing, and volume fraction (*F_v_*) of the particles. The effect of the second phase on the grain boundary per unit area (*P_z_*) is usually expressed by the following equation [23]:

(5) $$P_{Z}=\frac{3F_{v}\gamma }{2r}$$

here *γ* means the interface energy of the grain boundary per unit area. According to the microstructural observation, there is no precipitation of the second phase particles in the based alloy after solution treatment, while a large number of second phase particles were found in the modified alloy (Figure 4). If we take the modified alloy with solution processing at 1093 K for 1 h as an example, the related parameters of the secondary particles are shown in Table 6. It can be estimated that the pinning effect of the second relative unit area grain boundary in the modified alloy is 0.0165 MPa based on Equation (5), which is a result of the existence of the γ phase and Ni_16_Si_7_Ti_6_ particles. This pinning effect also plays a significant role in improving the thermal stability of the fine-grained structure in the Cu-15Ni-8Sn alloy during the solution treatment.

## 5. Conclusions

In this work, the microstructural features of the Cu-15Ni-8Sn alloy with and without adding Si and Ti were investigated. After the solution treatment at 1073~1113 K for 0.5~2 h, the initial (as-extruded) fine-grain structure with the grain size below 20 μm is able to be maintained in the Cu-15Ni-8Sn-0.3Si-0.1Ti alloy while grains are excessively coarsened in the Cu-15Ni-8Sn alloy. Based on the analysis of grain growth kinetics, the activation energy of the grain growth is increased from 156 kJ/mol to 353 kJ/mol, suggesting a significant improvement in the thermal stability of the fine-grained structure ascribed to additions Si and Ti. Owing to the solid solution Si in the γ phase, a large amount of γ phase would be retained after solution treatment. At the same time, Ni_16_Si_7_Ti_6_ particles are formed by introducing Si and Ti. Consequently, the grain boundary movement is severely hindered by two aspects: the pinning effect from the γ phase and Ni_16_Si_7_Ti_6_ particles, and the drag effect induced by solute Si and Ti atoms.

## Figures and Tables

**Table 1:** Chemical compositions of the as-cast alloys (wt.%).

Alloy Designation	Ni	Sn	Si	Ti	Cu
Cu-15Ni-8Sn (based alloy)	15.03	8.12	-	-	bal.
Cu-15Ni-8Sn-0.3Si-0.1Ti (modified alloy)	15.23	7.92	0.29	0.09	bal.

**Table 2:** Grain growth factor (*G_f_*), grain grow exponent (*n*), and activation energy (*Q*) for the studied alloys with a solution process of 2 h.

Alloy	*T* (K)	*G_f_*	*n*	*Q* (kJ/mol)
Cu-15Ni-8Sn	1073	6.70	3	156
	1093	7.76		
	1113	8.46		
Cu-15Ni-8Sn-0.3Si-0.1Ti	1073	0.30	10	353
	1093	0.36		
	1113	0.50		

**Table 3:** EDS results of corresponding positions in Figure 5 (wt.%).

Positions	Phases	Cu	Ni	Sn	Si	Ti
A	Ni_16_Si_7_Ti_6_	33.29	41.02	6.78	8.44	10.47
B	γ	74.7	17.07	9.62	0.35	-
C	γ	37.74	30.63	29.81	1.82	-

**Table 4:** EDS results of corresponding positions in Figure 7 (wt.%).

Positions	Phases	Cu	Ni	Sn	Si	Ti
A	γ	36.34	32.2	29.54	1.92	-
B	Ni_16_Si_7_Ti_6_	49.87	31.31	4.38	7.27	7.16

**Table 5:** The diffusion coefficients of different solutes in pure copper [22].

Solutes	*D* (10^−4^ m^2^/s)	*T*-Range (K)
Sn	0.84	1011–1321
Si	0.21	998–1173
Ti	0.693	973–1283

**Table 6:** Parameters of secondary particles in the modified alloy after the solution processing at 1093 K for 1 h.

*F_v_*	*r* (μm)	*γ* (J/m^2^) [24]	*P_z_* (MPa)
0.0075	0.444	0.65	0.0165

## Data Availability

The raw/processed data required to reproduce these findings cannot be shared at this time as the data also forms part of an ongoing study.
